# Investigation of a connection between abdominal wall defects and severity of the herniation in fetuses with gastroschisis and omphalocele

**DOI:** 10.1038/s41598-020-79599-y

**Published:** 2021-01-08

**Authors:** Natasha T. Logsdon, Carla M. Gallo, Luciano Alves Favorito, Francisco J. Sampaio

**Affiliations:** Urogenital Research Unit, State University of Rio de Janeiro, Rua Professor Gabizo, 104/201, Tijuca, Rio de Janeiro, RJ CEP 20271-320 Brazil

**Keywords:** Anatomy, Pathogenesis

## Abstract

Analyze the biometric parameters and the size (area) of abdominal wall defect (AWD) in fetuses with gastroschisis and omphaloceles and correlate them with the herniated internal organs. We studied 22 fetuses (11 with AWDs and 11 without anomalies). In all fetuses we evaluated the xiphopubic distance (XPD) and iliac crest distance (ICD). In fetuses with AWDs we dissected the abdominal wall and measured the width and length of the defect for calculating its area and studying the correlation between the size of the defect with the organs that were herniated. For statistical analysis, the Anova and Tukey post-test were used (p < 0.05). The XPD in the control group had mean of 4.2 mm (2.3–5.9; SD ± 1.11), while in the AWDs it was 4.2 mm (2.9–5.5; SD ± 0.98) (p = 0.4366). The ICD had mean values of 2.5 mm (1.6–3.4; SD ± 0.58) in the control group, and 2.3 mm (1.2–3.0; SD ± 0.56) in AWDs fetuses (p = 0.6963). The number of herniate organs do not have significant correlation with the area of the defect (r^2^ = 0.2504, p = 0.5068). There is no correlation between the size (area) of abdominal wall defects and the number of the internal organs that herniated. Therefore, the hole size is not a predictor of the severity of the gastroschisis or omphalocele.

## Introduction

Abdominal wall defects (AWDs) are common human birth anomalies with incidence of about 1 in 2000 newborns^1^. The AWDs that occur most commonly are gastroschisis and omphalocele^2^. Gastroschisis is a paraumbilical AWD associated with protrusion of the abdominal content through a defect, usually in the right side, without a covering membrane. Omphalocele is a AWD at the umbilicus, and the viscera outside the belly are covered by a membrane^2–4^. Patients with AWDs have an increased incidence of intrauterine growth restriction, and, therefore, the estimation of weight in fetuses with AWDs during gestational ultrasonography is more difficult than for normal fetuses^5–7^.

The abdominal wall develops from somitic and lateral plate mesoderm. Ventral body wall defects are originated from lateral plate mesoderm malformations^8^. The rectus abdominal muscle and rectus sheath are very important to physiological umbilical hernia closure during the abdominal wall development^9,10^. In omphalocele and gastroschisis, the rectus muscle was intact but inserted more laterally on the costal margins and xiphoid process^8^.

To our knowledge, there are no reports about the abdominal wall biometric parameters in human fetuses with AWDs. The objective of this study was to analyze the biometric parameters of the abdominal wall in AWDs fetuses and to compare them with the parameters of fetuses without anomalies. Also, we aimed to analyze the size (area) of abdominal wall defect in fetuses with AWDs and correlate it with the herniated organs.

## Methods

The fetuses used in this study (both Controls and with AWDs) were obtained from the Department of Pathology of the Fernandes Figueira Institute, Oswaldo Cruz Foundation, Ministry of Health, in partnership with our University, via an official Cooperation Term.

The study was approved by the Ethical Committee on Human Research—University Hospital of the State University of Rio de Janeiro (CEP / HUPE), with the number IRB: 2.770.641, CAAE: 89602318.4.0000.5259).

The study has also been registered in the Brazil Plataform, Ministry of Health, National Health Council, National Research Ethics Commission (CONEP) for studies with human beings. We confirm that all methods used in this paper were carried out in accordance with relevant guidelines and regulation.

We studied 11 human fetuses with AWDs, aged 15.3 to 27.4 weeks post conception (WPC), and 11 fetuses without anomalies (Controls) aged 13.2 to 18.8 WPC, during the period from March 2017 to February 2020. All fetuses were well preserved and have been demised due to spontaneous or induced abortion*.* The gestational age of the fetuses was determined in weeks post conception (WPC) according to the foot-length criterion, which is currently considered the most acceptable parameter to estimate the fetal gestational age^11–13^.

The fetuses were biometrically evaluated considered their total length, the crown-rump length (CRL) and the body weight.

**Figure 1:**
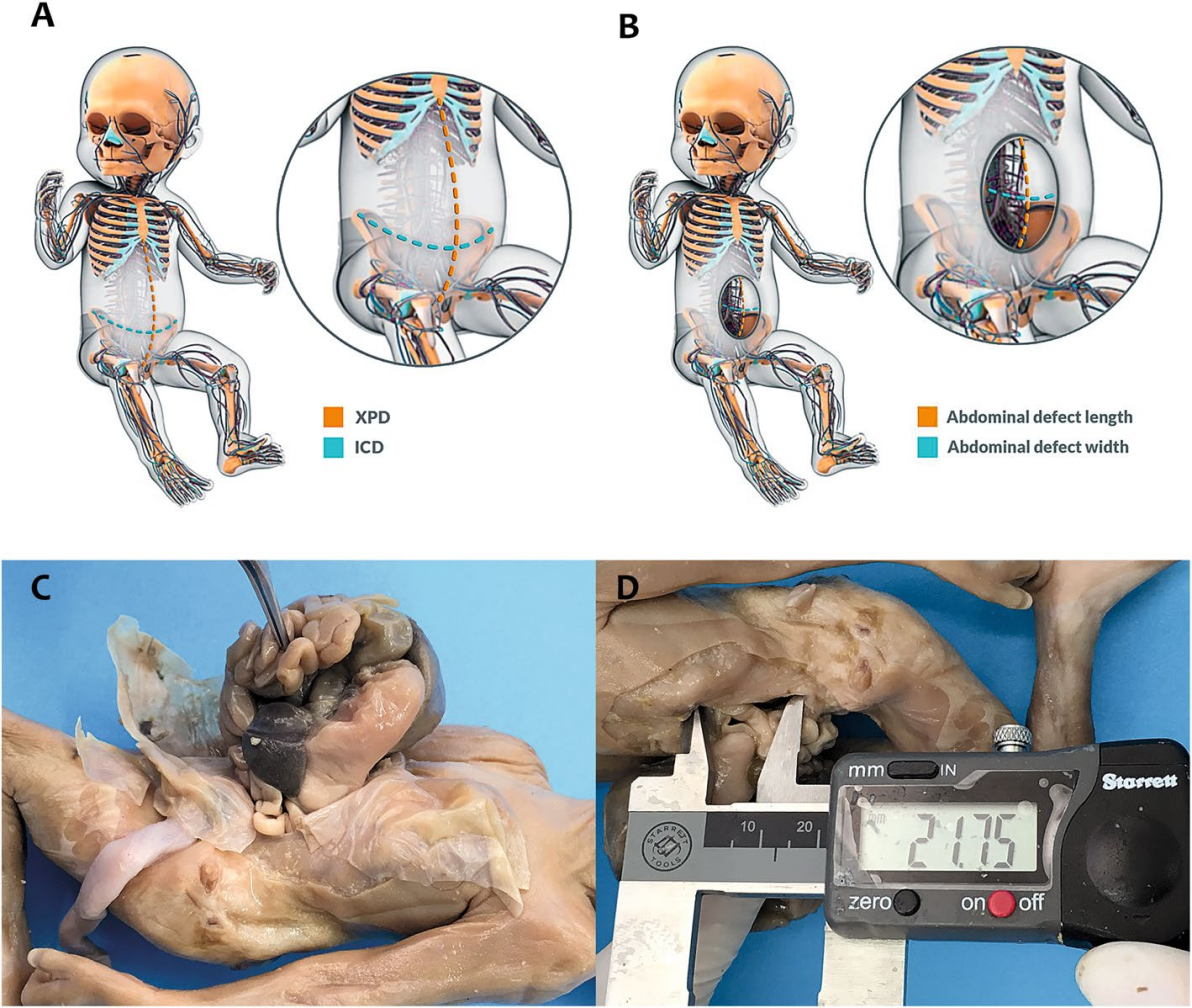
Morphometric evaluation of abdominal wall in controls and fetuses with abdominal wall defects (AWDs). (**A**) Schematic drawing showing the measurements of xiphopubic distance (XPD) and iliac crest distance (ICD); (**B**) schematic drawing showing the measurements of the length and the width of the defect in the abdominal wall in a fetus with AWD; (**C**) male fetus with 15 weeks post conception (WPC) with omphalocele, showing the dissected membrane and the defect in the anterior abdominal wall, with herniated abdominal organs; (**D**) male fetus with 15 WPC with a dissected omphalocele, showing the measurements of the abdominal wall defect with a digital pachymeter.

After these measurements, the fetuses were photographed and carefully dissected with the aid of a stereoscopic lens with 16–25× magnification. Two abdominal wall measures were recorded with a digital pachymeter in all 22 fetuses: xiphopubic distance (XPD) and the iliac crest distance (ICD) (Fig. 1A,B).

In the AWDs fetuses, we dissected and analyzed the abdominal wall defect as well as the number and kind of abdominal organs that were herniated. Figure 1C shows a typical aspect of an AWD fetus with omphalocele. The length and width of the abdominal wall defect were measured with a digital pachymeter (Fig. 1D), for calculating the area of the defect (Length × Width × 3.1416). The same observer performed all measurements.

### Statistical analysis

All parameters were statistically processed and graphically presented. Descriptive statistics were calculated and the values of age, weight, CRL, XPD and ICD are presented as means, followed by standard deviations (SD). The data were analyzed by the use of ANOVA and the Tukey post-test to compare variances among the groups. Differences were considered statistically significant when p-values were below 0.05. For the correlation of abdominal distances (XPD and ICD) and other variables, Pearson’s correlation coefficient was used considering r^2^ greater than 0.7 as strong correlation, while r^2^ between 0.4 and 0.7 reflected moderate correlation and r^2^ less than 0.4 reflected weak or very weak correlation. The statistical analysis was performed with the GraphPad Prism program (Version 8.0.1).

## Results

The statistical analysis of all fetal and abdominal biometric parameters is reported in Table 1.

**Table 1:** Statistical analysis of fetal and abdominal wall biometric parameters.

Parameter	Controls	AWDs	p Value
Age (WPC)	13.2–18.8 (mean = 15.7/SD ± 1.83)	15.3–27.4(mean = 19.5/SD ± 4.76)	0.0049
Weight (g)	58.0–344.0 (mean = 172.3/SD ± 94.67)	90.02728.0(mean = 786.6/SD ± 1056.1)	0.0227
CRL (cm)	9.0–19.0 (mean = 14.8/SD ± 3.12)	7.0–25.0 (mean = 15.9/SD ± 6.28)	0.0349
XPD (mm)	2.3–5.9 (mean = 4.2/SD ± 1.11)	2.9–5.5 (mean = 4.2/SD ± 0.98)	0.4366
ICD (mm)	1.6–3.4 (mean = 2.5/SD ± 0.58)	1.2–3.0 (mean = 2.3/SD ± 0.56)	0.6963

*AWDs *abdominal wall defects, *CRL *crown-rump length, *XPD *xiphopubic distance, *ICD *iliac crest distance.

The mean xiphopubic distance was 4.2 mm (2.3–5.9; SD ± 1.11) in the control group and 4.2 mm (2.9–5.5; SD ± 0.98) in the AWD fetuses, without significant differences between the groups (p = 0.4366). The mean distance between the iliac crests was 2.5 mm (1.6–3.4; SD ± 0.58) in the control group and 2.3 mm (1.2–3.0; SD ± 0.56) in AWD fetuses, without significant differences between the groups (p = 0.6963).

The size (length and width) as well as, the area of the hole in fetuses with AWDs, and the description of the abdominal organs that were herniated in these 11 cases are shown in Table 2. The hole in AWD had a mean length of 23.52 mm (13.46–35.14) and a mean width of 14.05 mm (7.33–25.83). The mean area of the hole was 1124.03 mm^2^ (379.30–2603.13).

**Table 2:** The table shows the description of herniated abdominal organs in the 11 fetuses with abdominal wall defects (AWD).

Fetus	Anomaly	Sex	Age (WPC)	Weight (g)	CRL (cm)	Abdominal defect length (mm)	Abdominal defect width (mm)	Abdominal defect area (mm^2^)	Herniated organs	Associated anomalies
1	O	M	15.3	96	10	13.46	8.97	379.30	Liver, small intestine, colon, appendix	Left pelvic kidney, bilateral hydronephrosis (UPJ stenosis)
2	G	F	15.4	90	7	17.00	7.5	400.55	Small intestine, L-lung, heart, spleen, liver, stomach, pancreas, large intestine (except sigmoid)	Facial malformation
3	G	F	16.1	154	11	22.85	10.17	730.06	Liver, spleen, stomach, small intestine, large intestine, R‐adrenal gland	Encephalocele
4	G	F	16.3	142	14	26.22	12.66	1042.84	Liver, small intestine, stomach, spleen, asce nding colon, transverse	None
5	G	M	16.3	178	14	20.49	11.98	771.17	Small intestine, L-colon, descending sigmoid	Cleft lip and malformation of upper limbs and face
6	G	F	16.7	210	15	21.01	7.33	483.82	Liver, spleen, small intestine, large intestine (except cecum), R-­adrenal gland	None
7	O	M	18.4	306	16	21.17	13.33	886.55	Liver, spleen, caecum with appendix, small intestine, stomach, ascending colon, common hepatic flexure, esophageal-–gastric junction	Left lower limb agenesis
8	G	F	19.8	256	13	35.14	23.58	2603.13	Liver, small intestine, large intestine, stomach, spleen, L-lung	Facial malformation and upper limbs
9	O	M	26.1	1988	25	22.12	16.06	1116.04	Liver, small intestine, stomach, spleen	Bilateral renal agenesis
10	G	F	26.2	2728	24.5	31.46	17.15	1695.02	Liver, heart, L-lung, spleen, intestinal loops, ovaries, stomach, kidneys, adrenals, uterus, uterine tubes	None
1 1	G	F	27.4	2504	25	27.80	25.83	2255.90	Liver, stomach, small intestine, large, spleen, L-ki dney	Discoid kidney

Age in weeks post-conception (WPC); weight in grams (g), crown-rump length in centimeters (cm); abdominal wall defects (length and width) in millimeters (mm), area of abdominal defect (hole) in square millimeters (mm^2^). *G *gastroschisis, *CRL *crown-rump length, *O *omphalocele, *UPJ *ureteropelvic junction.

The linear regression analysis indicated that the XPD in the Control group (r^2^ = 0.6837; p = 0.0017) and in the AWD group (r^2^ = 0.6106; p = 0.0045) increased significantly and positively with fetal age (p < 0.0001) (Table 3). The ICD also increased significantly and positively with fetal age in the Control group (r^2^ = 0.466; p = 0.0255) and in the AWD group (r^2^ = 0.6742; p < 0.0019) (Table 3). The XPD in the Control group (r^2^ = 0.8161; p = 0.0001) and in AWD group (r^2^ = 0.4919; p = 0.0162) increased significantly and positively with fetal weight (Table 3). The ICD also increased significantly and positively with fetal weight in the AWD group (r^2^ = 0.5453; p = 0.0094), but only the Control group had strong correlation with fetal weight (control group: r^2^ = 0.8161; p = 0.0001) (Table 3).

**Table 3:** The table shows the linear regression analysis (correlation strength: strong, moderate and weak) in the 22 fetuses: 11 with abdominal wall defects (gastroschisis and omphalocele); and 11 without anomalies (controls).

Linear regression	r value	p value	Correlation strength
XPD × Age – Controls	r^2^ = 0.6837	p = 0.0017	Moderate
XPD × Age – AWDs	r^2^ = 0.6106	p = 0.0045	Moderate
ICD × Age – Controls	r^2^ = 0.4666	p = 0.0295	Moderate
ICD × Age – AWDs	r^2^ = 0.6742	p < 0.0019	Moderate
XPD × Weight – Controls	r^2^ = 0.8161	p = 0.0001	Strong
XPD × Weight – AWDs	r^2^ = 0.4919	p = 0.0162	Moderate
ICD × Weight – Controls	r^2^ = 0.8161	p = 0.0001	Strong
ICD × Weight – AWDs	r^2^ = 0.5453	p = 0.0094	Moderate
XPD × ADA	r^2^ = 0.5956	p = 0.0054	Moderate
Age × ADA	r^2^ = 0.4534	p = 0.0231	Moderate
ICD × ADA	r^2^ = 0.5534	p = 0.0087	Moderate
Herniated Organs × ADA	r^2^ = 0.2504	p = 0.5068	Weak

*Age *  fetal age in weeks postconception, *XPD * xiphopubic distance, *AWD * abdominal wall defects, *ICD * iliac crest distance, *ADA * abdominal defect area.

The linear regression analysis indicated that the XPD in the Control group (r^2^ = 0.7394; p < 0.0007) and in the AWD group (r^2^ = 0.4823; p < 0.0177) increased significantly and positively with fetal CRL. Also, the ICD in the Control group (r^2^ = 0.5501; p = 0.0090) and in the AWD group (r^2^ = 0.6560; p = 0.0025) increased significantly and positively with CRL (Table 3).

The linear regression analysis indicated that the abdominal defect width increased significantly and positively with fetal weight (r^2^ = 0.3883; p = 0.0406) and with the fetal age (r^2^ = 0.3999; p = 0.0368), nevertheless, the abdominal defect length do not have significant correlation with the fetal weight (r^2^ = 0.1998; p = 0.1681) and fetal age (r^2^ = 0.2201; p = 0.1454) (Table 3).

The number of herniate organs through the abdominal wall defect do not have significant correlation with the length (r^2^ = 0.1348; p = 0.2668) and width (r^2^ = 0.01768; p = 0.6967) of the defect, nor with the area of the defect (Table 3).

**Figure 2:**
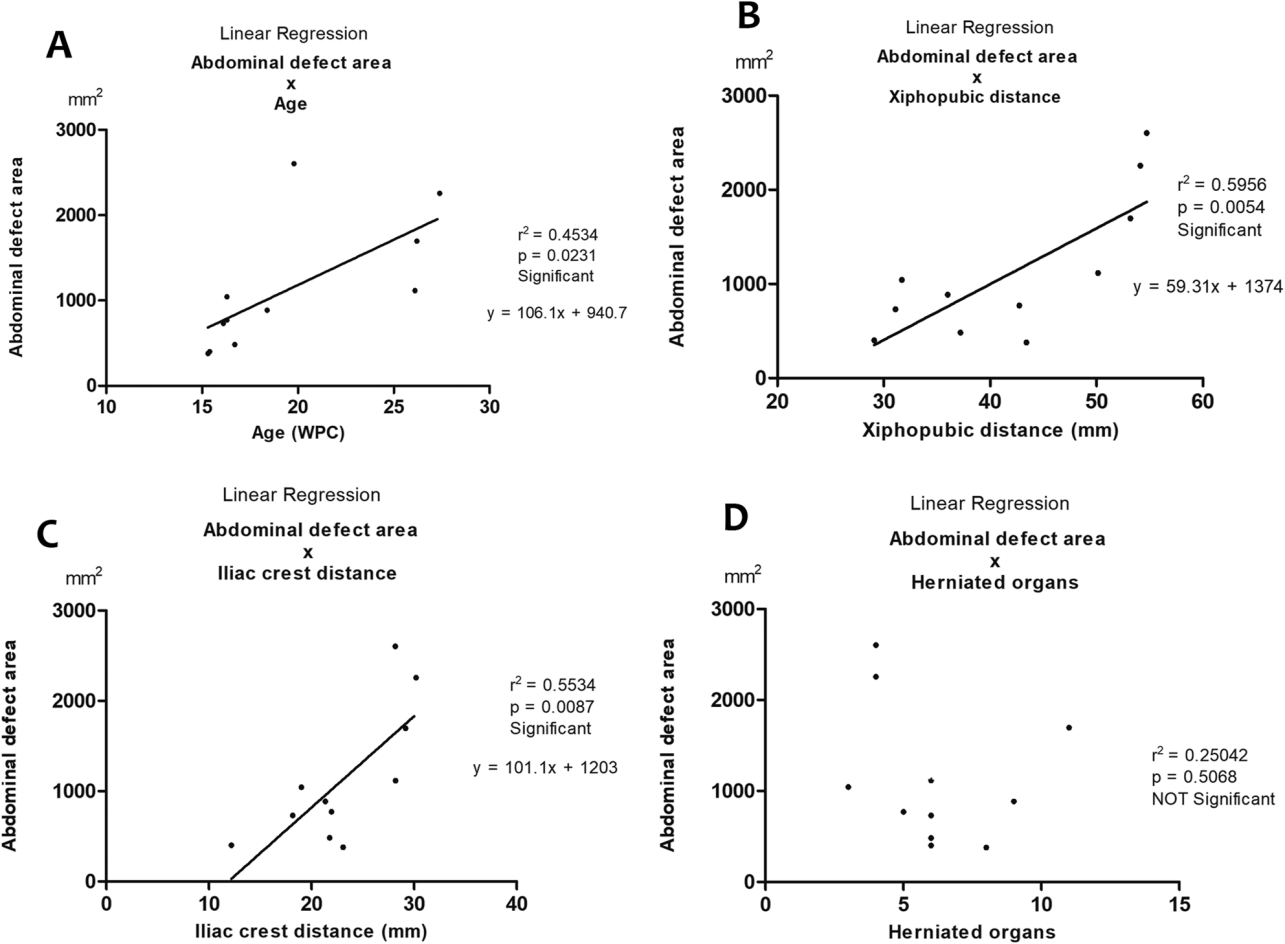
(**A**) Linear regression analysis of Abdominal Defect Area (ADA) versus Fetal Age showing that the ADA increased significantly and positively with fetal age (r^2^ = 0.4534; p = 0.0231); (**B**) linear regression analysis of ADA versus Xiphopubic Distance (XPD) showing that the ADA increased significantly and positively with the XPD (r^2^ = 0.5956; p = 0.0054); (**C**) linear regression analysis of ADA versus Iliac Crist Distance (ICD) showing that the ADA increased significantly and positively with the ICD (r^2^ = 0.5534; p = 0.0087); (**D**) linear regression analysis of ADA versus Number of Herniated Organs showing that the area of abdominal defect (hole) did not have significant correlation with the number of herniated organs (r^2^ = 0.25042; p = 0.5068).

Figure 2 shows the graphics of the following linear correlations: Abdominal Defect Area (ADA) vs. Fetal Age, Abdominal Defect Area vs. Xiphopubic Distance, Abdominal Defect Area vs. Iliac Crest Distance and Abdominal Defect Area vs. Herniated Organs.

## Discussion

During the 4th WPC, the abdominal wall is formed in the craniocaudal and mediolateral directions^14^. From the 6th WPC, there is physiological herniation of the liver and midgut due to inadequate space in the abdominal cavity for the rapidly growing of the medium intestine^15,16^. The midgut completed its rotation and returns to the abdominal cavity at the 10th WPC. Omphalocele is characterized by the failure of the physiological hernia to return to the abdominal cavity^17^. On the other hand, the cause of gastroschisis is not completely elucidated, but there is evidence of an abnormality in the formation and development of the ventral body wall during embryogenesis, resulting mainly in bowel herniation^18^. Thus, the origin of this defect is different from that of omphalocele^19,20^.

Patients with omphalocele have a high prevalence of associated anomalies, while gastroschisis is associated with malformations outside the gastrointestinal tract in around 10% of the cases, and with abnormalities related to the gastrointestinal tract in up to 25% of cases^21–23^. Although our AWDs sample was small, from 8 cases of gastroschisis studied, we found 5 cases (62.5%) with anomalies not associated with the gastrointestinal tract, such as craniofacial malformations, limb agenesis and kidney anomalies.

The AWDs diagnosis can be easily performed by ultrasound around the 11th to 12th WPC^6,7^. Growth restriction is an important predictor of perinatal morbidity and mortality in gastroschisis and omphalocele, so the accurate estimation of fetal weight is important to guide the management of fetuses with AWDs^24,25^. Estimation of weight in fetuses with AWD was very difficult and no formula used in ultrasonography during the gestational period has yet shown good accuracy^6,7^. In AWDs fetuses, the abdominal circumference measurements by ultrasonography may overestimate the weight^6,7^.

The AWDs fetuses in our sample had higher weight and CRL when compared to controls, but in this group we had 3 fetuses with more than 26 WPC, explaining the significant differences in weight and CRL compared with the control group. The analysis of the linear regressions indicated interesting findings when comparing the abdominal wall parameters with fetal weight and crown-rump length. The biometric parameters of the abdominal wall had strong correlation with fetal weight and crown-rump length only in the fetuses of the control group. These findings support the association of AWDs with intrauterine growth restriction during the gestational period.

In fetuses with defects in the abdominal wall, the organs tend to protrude out through the abdominal hole^26^. In most cases, two or more organs (e.g., liver, intestines and stomach) are herniated^27,28^. As expected, we have observed this condition in most of our fetuses. However, despite being rarely found in these cases, we found a herniated spleen in 9 of 11 fetuses. In our sample, the organs most often herniated were liver and small intestine (91%), large intestine (82%), followed by stomach and spleen (73%). The evisceration only of the intestine classifies gastroschisis as simple, while the evisceration of other organs classifies it as complex^23–26^. Studies have shown that this complex condition is correlated with an increase in the mortality rate^27–30^.

Our findings suggest that the area of the abdominal wall defect (hole) in gastroschisis and omphalocele was not a predictor of the number of herniated abdominal organs. The linear regression analysis (Table 3 and Fig. 2) confirmed this information, showing that the number of herniate organs through the abdominal wall defect do not have significant correlation with the area of the abdominal wall defect.

Steven et al. (2019), in a recent multicentric study with 274 omphaloceles patients shows that the defect size is an independent predictor of neonatal morbidity and mortality, nevertheless, they do not performed the abdominal wall defect measurements; they only classified the defect as small, medium, large, giant and unknown^31^.

Our paper is the first to report correlations of the abdominal wall measurements with fetal age, weight and CRL in AWD fetuses. For the first time we also analyzed the measurements of the abdominal wall defects in human fetuses with gastroschisis and omphalocele, and correlated it with the xiphopubic and iliac distances, as well as with the herniated organs.

An important limitation of our study should be mentioned: the sample size was small, however, fetuses with gastroschisis and omphalocele are very rare and observations of a small sample are still relevant.

## Conclusion

There is moderate correlation between the xiphopubic distance and the area of abdominal defect, as well as between the iliac crest distance and the area of the abdominal defect.

There is no correlation between the size (area) of abdominal wall defect and the number of the internal organs that herniated. Therefore, the size of the hole is not a predictor of the severity of the gastroschisis or the omphalocele.

## Ethical approval

This study was carried out in accordance with the ethical standards of the hospital’s institutional committee on human experimentation. (IRB: 2.770.641, CAAE: 89602318.4.0000.5259).

## Abbreviations

ADA: Abdominal defect area
AWD: Abdominal wall defect
AWDs: Abdominal wall defects
CAAE: Certificate of ethical appreciation presentation
CEP: Ethical Committee on Human Research
CNPQ: National Council for Scientific and Technological Development
CONEP: National Research Ethics Commission
CRL: Crown-rump length
FAPERJ: Rio de Janeiro State Research Foundation
HUPE: Pedro Ernesto Universitary Hospital
ICD: Iliac crest distance
IRB: Institutional Review Board
mm: Millimeters
mm^2^: Square millimeters
p: P-value
r2: Pearson correlation coefficient
SD: Standard deviation
Vs.: Versus
WPC: Weeks post conception
XPD: Xiphopubic distance
